# Recent Advances in Chiral Nematic Structure and Iridescent Color of Cellulose Nanocrystal Films

**DOI:** 10.3390/nano6110213

**Published:** 2016-11-14

**Authors:** Derek G. Gray

**Affiliations:** Department of Chemistry, McGill University, Montreal, QC H3A 2A7, Canada; derek.gray@mcgill.ca; Tel.: +1-514-398-6182

**Keywords:** cellulose nanocrystals, chiral nematic, polarized light microscopy

## Abstract

One unique property of cellulose nanocrystals (CNC) is their property of forming suspensions with chiral nematic order. This order can be preserved in films cast from the suspensions, raising the possibility of applications as photonic materials and templates. However, it has proved difficult to generate uniform, well-ordered chiral nematic materials from CNC. Recently, the importance of kinetic arrest due to gel formation in the later stages of evaporation has been recognized as a key step in film formation. In this brief review, recent developments regarding the structure of chiral nematic suspensions and films as monitored by polarized light microscopy are outlined, and attention is drawn to the importance of shear forces on the self-organization process.

## 1. Introduction

Cellulose nanocrystals (CNC) and other forms of nanocellulose are gaining recognition as an important new family of sustainable materials with a wide range of potential applications [1,2]. A surprising property of cellulose nanocrystal suspensions was that the rod-like nanocrystals formed a chiral nematic phase above a critical concentration [3], and that this suspension dried to form an iridescent film with the optical properties of a chiral nematic liquid crystal [4]. Chiral nematic (cholesteric) liquid crystals show extremely high optical rotatory power, and reflect one hand of circularly polarized light in a narrow wavelength band. The other hand of circularly polarized light is transmitted without change. The wavelength of the reflected band changes with viewing angle. These properties were explained by de Vries [5] on the basis of a helicoidal arrangement of birefringent layers. He predicted that the wavelength of reflected circularly polarized light, λ, would depend on the pitch, *P*, of the chiral nematic structure, λ = *nP* where *n* is the mean refractive index of the chiral nematic phase. The handedness of the reflected light depends on the handedness of the chiral nematic structure. This relation between wavelength and pitch holds for samples viewed along the chiral nematic axis (i.e., normal to the sample surface). The wavelength reflected from a given sample becomes shorter at oblique angles, following the approximate relationship λ = *nP*cos*θ*, where *θ* is the angle between the normal to the surface and the viewing angle. Thus, a sample with a black background that reflects red light when viewed at right angles to the surface would appear green when viewed at 45°. In typical low molecular weight liquid crystals, the chiral nematic structure is made up of molecules such as cholesterol esters, but similar optical properties have been observed for films made from CNC suspensions.

While many structural features govern the iridescent appearance, the primary variable governing the structural color of the film is the helicoidal pitch, *P*, of the chiral nematic organization. In early work, the pitch was observed to decrease (move towards the blue end of the spectrum) when electrolyte was added to the suspension [4,6]. Beck and coworkers subsequently found that ultrasonication of the precursor suspension moved the reflection band of CNC suspensions and films to longer wavelengths [7], thus demonstrating that iridescent films of any desired wavelength can be prepared from CNC suspensions by combining electrolyte addition with ultra-sonication.

The relationship between the chiral nematic pitch in suspension and the properties of individual cellulose nanoparticles must depend on some chiral component of their shape, surface charge or molecular structure, but details remain obscure. Nevertheless, the changes in pitch with concentration are essentially reversible over a wide concentration range. What is now clear is that kinetic factors come into play as the concentration of the suspension increases in the final stages of forming a dry film. Processes discussed below result in a “freezing-in” of the chiral nematic structure, and hence play a key role in the final value of *P* in the film. This freezing-in of chiral nematic order has previously been proposed as occurring during the preparation of chiral nematic films from concentrated solutions of cellulose derivatives [8]. All evidence to date is in accord with the postulate of a left-handed helicoidal arrangement of CNC in the films [9]. Understanding the factors that control *P* is crucial for proposed applications of CNC and CNC-templated materials as photonic components. In this review we only consider the properties of aqueous suspensions of CNC. The many interesting organic and inorganic chiral nematic materials prepared from CNC and CNC templates are not considered here.

## 2. Overview of Drying Process

Unless otherwise stated, aqueous suspensions of conventional sulfate half-ester stabilized CNC are considered here. The increase in nanocrystal concentration from dilute suspension to solid film passes through several stages.

1. A dilute isotropic suspension in which individual CNC, normally stabilized by anionic sulfate half ester or carboxyl groups on their surfaces, are in Brownian motion, giving a clear slightly viscous fluid when the suspension is stable.
2. As the concentration of CNC is increased, an ordered chiral nematic phase starts to form. The ordered phase often initially appears as “tactoids”, droplets of anisotropic phase surrounded by the isotropic phase. Note that in this biphasic region, the concentration of CNC in the continuous isotropic phase is only slightly lower than in the anisotropic phase [6], and that the tactoids readily aggregate to form a continuous anisotropic phase. In principal, *P* remains constant across the biphasic region.
3. As the concentration is increased beyond the two-phase region, the suspension becomes a cloudy viscous fluid that appears almost completely anisotropic by polarized light microscopy. The pitch, *P*, decreases monotonically as the concentration of CNC increases.
4. Depending on the dimensions and charge of the CNC, a concentration is reached where the suspension starts to form a gel, inhibiting the further decrease in *P*.
5. Eventually, the gel may lose more water, trapping the chiral nematic organization in the glassy state.

**Figure 1:**
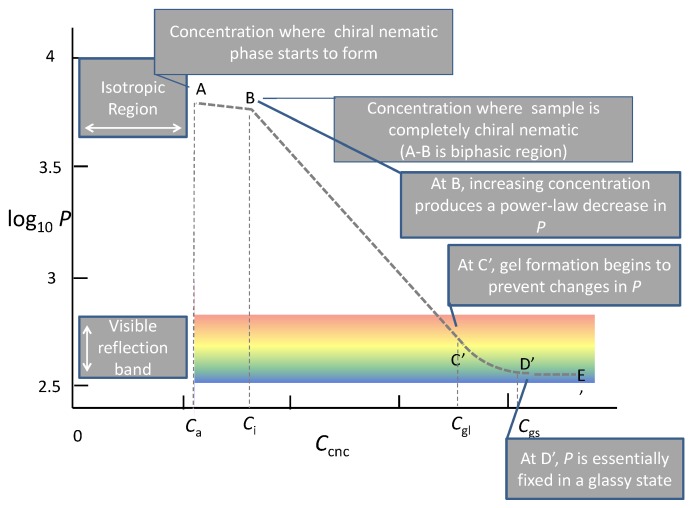
Schematic illustrating the observed dependence of the chiral nematic pitch, *P*, as a function of cellulose nanocrystals (CNC) concentration, *C*_cnc_, as water evaporates to form a solid film. The rainbow area represents the range of *P* values that reflect visible light. *C*_a_ and *C*_i_ represent critical concentrations for the onset and completion of the chiral nematic phase separation, respectively, and *C*_gl_ and *C*_gs_ represent concentrations for gel and solid glass formation, respectively (adapted from [10]).

The drying stages are represented schematically in Figure 1. The final pitch of the CNC film will depend on where along the line A–E the structure of the suspension is frozen.

## 3. Kinetic Factors that Control *P* during Evaporation

The role of jamming in controlling chiral nematic pitch as CNC suspensions dry has been postulated in the literature [10,11,12]. However, the concentration at which the onset of gelation occurs is difficult to measure independently. Rheological measurements provide a guide [13], but, as both ordered phase formation and gelation depend on CNC free volume and orientation, establishing a clear cut correlation between variations in *P* and rheological properties is challenging. However, adding glucose to CNC suspensions provided evidence for the importance of jamming in determining *P* in films. Glucose is a relatively inert sugar, similar to the repeat units in cellulose. The addition of *d*-(+)-glucose has no significant effect on the phase equilibrium for a given overall CNC concentration. However, the addition of *d*-(+)-glucose causes the pitch of chiral nematic long-pitch *suspensions* to decrease, but shifts the (much shorter) pitch of the *dry films* to longer values, thus moving the iridescent reflection of the films to the red end of the spectrum [10]. These results support the proposition that two distinct mechanisms operate as chiral nematic films form by evaporation, with the addition of glucose enhancing the tendency to twist at a given CNC concentration, but also enhancing the tendency to gel, and hence inhibiting further decrease in pitch as the suspension dries.

The self-assembly of chiral nematic structures was followed by controlled evaporation of water from CNC suspensions prepared by sulphuric acid hydrolysis of a eucalyptus sulphite wood pulp [14]. The authors proposed three stages of evaporation. The first stage was evaporation, during which the concentration increased, but the suspension remained isotropic. The next stage the authors called the gel phase, where finger-print patterns were observed on the upper boundary of the suspension-air interface. The decrease in spacing with evaporation time suggests that the CNC suspension was in the anisotropic rather than the biphasic state. (The term “tactoid” is usually reserved for droplets of anisotropic phase dispersed in the isotropic phase.) In the third phase, the sample became coloured due to the reflection of circularly polarized light. The wavelength of the reflected light decreased as evaporation proceeded. The rate of decrease with time was non-uniform, slowing down as evaporation proceeded, presumably due to a decrease in water activity, and then speeding up as the film approached dryness. This behaviour is essentially in accord with the scheme in Figure 1, the number of stages depends on the assumed starting point.

New light on the complex physical chemistry of CNC phase separation, chiral nematic pitch and kinetic arrest was recently provided by Honorato-Rios et al. [15]. They prepared CNC with two different surface charges from cotton, and measured the phase compositions and chiral nematic pitch across the biphasic region for several added salt concentrations. The pitch was longer for the low charge CNC, and decreased for both samples when salt (<2 mM) was added. Addition of salt also decreased the difference in concentration between isotropic and chiral nematic phases in the two-phase region. One conclusion of experiment and Monte Carlo modelling is that added salt causes the isotropic-chiral nematic phase transition to lose its first-order character, thus making it difficult to establish the phase diagram. (A similar situation occurs for the (hydroxypropyl)cellulose/water system, where as far as is known, no clear phase separation has been observed) Another puzzling observation is the appearance of a three-phase region for 9 wt % concentrations of the low-charge sample. The effect of surface charge on kinetic arrest was addressed by considering appropriate rheological measurements [15]. 

The early onset of kinetic arrest as the concentration of CNC increases may prevent the chiral nematic pitch decreasing to values where iridescence is observed. The onset of gelation (“kinetic impairment”) and glass formation (“kinetic arrest”) occur when the mobility of CNC in the suspension is inhibited, either by geometric factors (“entanglement”) or by loss of electrostatic or electrosteric stabilization of the CNC suspension. However, the formation of stable gels from CNC suspensions may be desirable for some applications. For example, thixotropic gels of CNC in glycerol were prepared by careful evaporation of water from dilute glycerol-water suspensions of acid-form CNC [16]. The much lower volatility of glycerol compared to water facilitated the evaporation of water and the increased concentration in glycerol. The authors attributed the onset of gelation to the solvolysis and removal of stabilizing sulfate half-ester groups on the CNC surface. In a recent publication, Bruckner et al. [17] used a similar difference in volatility to prepare stable CNC suspensions in formamide, N-methyl formamide and dimethyl formamide (DMF), and compared their behavior with that in water. The suspension in DMF showed a sharp kinetic arrest at a CNC concentration of only 2 wt %, a much lower concentration than those for the other solvents. A possible explanation was proposed in terms of the relatively low dielectric constant for DMF compared to the other suspending media. The authors also reported intriguing new information on the effect of the dielectric properties of the suspending media on the observed values of the chiral nematic pitch [17].

## 4. Carboxyl-Stabilized CNC Suspensions and Films

Most research on CNC has focused on nanocrystals prepared by sulfuric acid hydrolysis that are stabilized by surface sulfate half-ester groups. However, cellulose nanocrystals stabilized by surface carboxyl groups have also been prepared by a range of oxidative reactions. Araki et al. [18] found that cotton cellulose, subjected to acid hydrolysis by HCl (rather than H_2_SO_4_) followed by TEMPO (2,2,6,6-tetramethylpiperidine-1-oxyl catalysed) oxidation gave carboxyl stabilized CNC (cCNC) suspensions that showed the typical finger-print patterns characteristic of chiral nematic order with line spacings of 7 µm at 9.5 wt % total concentration. Subsequently, a simple one-step method of isolating nanocrystals by ammonium persulfate oxidation was discovered by Luong and coworkers [19,20]. The carboxyl-stabilized CNC were found to form chiral nematic suspensions and films, but with pitch values longer than those required for iridescence [21]. Typical finger-print patterns were observed for acid-form cCNC suspensions of approximately 5 wt % concentration. At this concentration, the suspensions were biphasic, with values for *P* of around 12–20 µm. The suspensions seemed to be essentially anisotropic at 9 wt % cCNC. No systematic differences were observed for cCNC prepared from cotton or wood pulp, or for cCNC in which the carboxyl groups had H^+^ or Na^+^ counterions. On drying, the films scattered light, but showed no evidence of iridescent color. Evidently the lower colloidal stability of cCNC allowed gelation of the suspension before *P* decreased to values where visible light is reflected. However, there is clearly a need to confirm this by further observations on purified and well-characterized cCNC suspensions.

## 5. Liquid Crystalline Textures Observed by Polarized Light Microscopy

The local orientation of the chiral nematic director is most readily observed by polarized light microscopy. The orientation of the director, and the disclination lines and planes where the director orientation is discontinuous make up the so-called “texture” of the suspensions and films [22]. (Care must be exercised in employing alternative scattering methods to determine orientation and order, as such methods often sample large volumes with non-uniform orientations, or small volumes that are not characteristic of the overall sample structure.) The observed texture depends on the concentration and initial state (isotropic, biphasic or chiral nematic) of the suspension, together with the rate and mode of evaporation, the shear history of the sample, and its mode of preparation for microscopy. For well-stabilized de-ionized aqueous CNC suspensions, starting from a concentration in the isotropic or biphasic range, the sequence of textures is well-established [3]. As mentioned above, tactoids of fluid chiral nematic phase are observed to form in the isotropic phase. These display the finger-print texture of uniformly spaced light and dark rings, with spacing of *P*/2. Each droplet must include at least one disclination [22]. The interfacial tension between the isotropic and chiral nematic phases is very low [23], and as the CNC concentration increases, the droplets grow, become distorted and coalesce under gravity.

The texture observed between microscope slides and cover glass depends on many factors that include the initial concentration of the CNC suspension, the shear forces unavoidably applied when depositing the suspension onto the microscope slide, and when pressing the cover slip onto the suspension, the thickness of the sample, the rheology of the sample and the time allowed for the sample to relax. For long pitch samples, extensive regions of finger-print texture may be observed by focussing towards the mid-plane of the sample. When evaporation is prevented, the finger-print line spacing is relatively constant across the sample, but dark disclination lines often separate regions with different director orientations. With well-sealed liquid crystalline samples, the disclination lines often disappear with time, giving mono-domains with uniform director orientation. Magnetic fields may be used to generate well-oriented uniform suspensions and films [24,25,26].

## 6. Polarized Light Microscopy of Solid CNC Films

The pitch and texture kinetically trapped in solid CNC films are clearly dependent on a large number of ill-understood factors. The source of the observed chirality must depend on the chiral structure of the individual nanocrystals, but the mechanism is still contentious and outside the scope of this review. What is clear is that the initial nanocrystal concentration, the rate of evaporation, the temperature, the dimensions and surface charge of the nanocrystals, the ionic strength all influence the chiral nematic pitch, and the orientation and distribution of the chiral nematic axes in the sample govern the liquid crystal texture observed by polarized light microscopy. Not surprisingly, the textures of CNC films prepared by evaporation from the surface of CNC suspensions are often ill-defined, with many disclinations, in line with the rather broad, weak, circularly-polarized reflectance spectra observed for many samples. The most intense reflection colors would be expected for films with planar texture where the optical axis of the chiral nematic structure is perpendicular to the plane of the film. Recently, the structure and optical properties of dry films prepared from well-stabilized CNC suspensions were examined in an elegant series of experiments by Dumanli et al. [27]. The films displayed domains of bright uniform iridescent colours due to the reflection of left-handed circularly polarized light. Electron microscopy of the cross-section of the films showed the helicoidal arrangement of the nanocrystals, with pitch values in good agreement with those derived from the reflection spectra.

**Figure 2:**
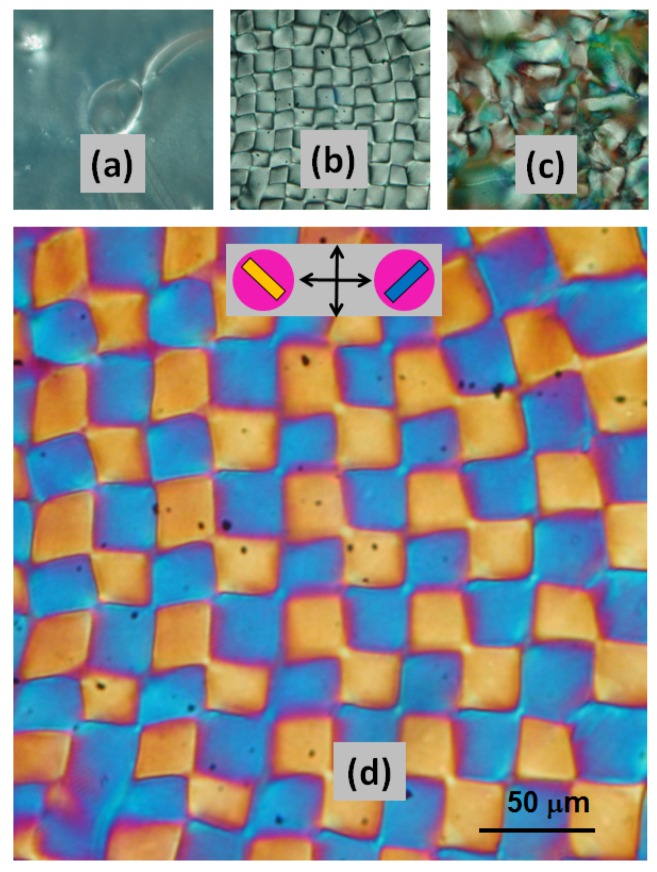
Polarized light images of CNC films (area 300 µm × 300 µm). (**a**) 12 µm; (**b**) 24 µm; (**c**) 36 µm thick films between crossed polars; (**d**) shows the parabolic focal conic image (**b**) magnified between crossed polars with 530 nm red wave plate. Direction of crossed polars shown by arrows; orientation of CNC shown by insert on (**d**). Figure 2b,d adapted from [28].

Under some circumstances, the disclinations form a regular pattern that may be trapped in the dry film [28]. An example is shown in Figure 2, where the textures of films cast from different amounts of a CNC suspension show an effect due to final film thickness. The thin film (Figure 2a) shows a relatively featureless texture, where the chiral nematic axis is perpendicular to the plane of the film. The square checkerboard pattern corresponds to a parabolic focal conic texture (Figure 2b,d), where the length of sides of the square elements is close to the thickness of the film. The texture of the thickest sample (Figure 2c) resembles the textures widely observed for CNC films, and indicates a more random director orientation.

Most solid CNC films showing visible reflection bands have been prepared by evaporation of water from aqueous suspensions in shallow, flat open containers, usually made from polystyrene. The dry film adheres fairly weakly to polystyrene, allowing films with areas of many cm^2^ to be detached without cracking. However, the properties at the edges of such films differ from that in the middle. The films are thicker, the reflection band wavelengths are usually longer, and the texture is also different at the edge. A related behaviour is observed for small droplets evaporating on a plane surface. Again, the properties at the edge of the residual films differs from that in the middle, in this case because of concentration-driven mass transfer of CNC to the evaporating edge of the droplet, resulting in a raised ring at the edge [29]. This behaviour results from the strong pinning of the contact line at the edge of the droplet. If the contact line is weakly pinned, and can retreat in a stick-slip motion as droplet evaporation proceeds, then a beautiful texture showing concentric rings of increasing thickness is generated [30].

## 7. Topography of Solid CNC Film Surfaces

Evaporation from the upper surface of CNC suspensions often generates dry films whose topology mirrors the orientation and textures observed by polarized light microscopy. This was observed by atomic force microscopy (AFM) of parabolic focal conic CNC films [28]. The topology reflects the anisotropic shrinkage of the chiral nematic structure. The rippling observed at the surface of finger-print texture [28,30] results from the tendency of ordered regions to shrink more readily in a direction perpendicular to the mean CNC orientation than parallel to the CNC. A relevant theoretical interpretation for rippling at chiral nematic free surfaces has been presented by Rofouie et al. [31].

## 8. Shear Effects on Texture and Surface Topography

**Figure 3:**
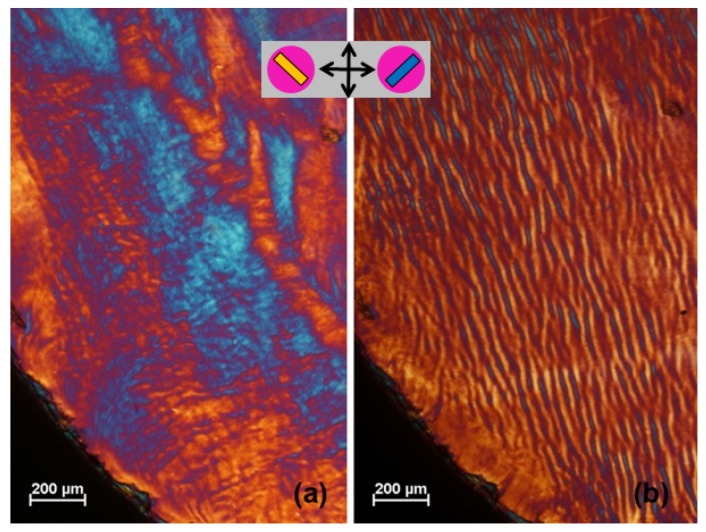
Effect of shear relaxation on texture of 6 wt % carboxyl stabilized CNC (cCNC) aqueous suspension, viewed by polarized light microscope between crossed polars with 530 nm red wave plate, oriented as in insert. (**a**) As initially prepared between microscope slide and cover glass; (**b**) after relaxing for 3 min after applying a left-to right shearing motion to cover glass.

The texture of CNC films prepared from chiral nematic suspensions is sensitive to shear applied to the sample during the drying process [13,32]. A complicating factor is the effect of shear relaxation on the texture. Visco-elastic suspensions often display a banded texture on relaxing after shear. The bands are usually transverse to the direction of shear, and their spacing is around an order of magnitude greater than that of finger-print lines, and lack the uniformity of the latter .An example of banded texture is shown in Figure 3 for a suspension of carboxyl-stabilized cCNC. Squeezing a sample of the viscous suspension between microscope slide and cover glass gives birefringent areas oriented essentially at random (Figure 3a). A shearing motion was then applied to the sample by sliding the cover glass relative to the microscope slide. The band texture that developed at right angles to the shearing motion after 3 min is shown in Figure 3b. The appearance of the lines is quite different from the chiral nematic finger-print pattern. The lines in the banded texture are discontinuous: they disappear with time in the fluid state as the effects of shear relax, and the spacing is typically much larger than the *P*/2 line spacing normally observed for finger-print texture.

In a fascinating study motivated by the iridescent properties of some tulip petals [33], shear-cast cellulosic films formed band structures with matching surface corrugations normal to the shear direction, and also formed secondary corrugations corresponding the chiral nematic pitch. The combination of controlled surface topography and controlled chiral nematic pitch opens up novel photonic applications.

A comparison of films from sheared and un-sheared CNC suspensions showed differences in internal structure but similar reflection properties. The broad reflection bands observed spectroscopically were attributed to the polydispersity in CNC dimensions [34]. In a further examination of the effect of shear on texture for concentrated CNC suspensions, a transitory nematic-like texture appeared to follow an initial brief banded texture as the sample relaxed to the normal chiral nematic state. A novel twist-bend mechanism was proposed for the transition between nematic and chiral nematic order [35].

## 9. Conclusions

Clearly, progress has been made recently in understanding some of the factors that influence the optical properties of chiral nematic films prepared from cellulose nanocrystal suspensions. However, the understanding remains qualitative, and the interplay between chiral interactions and kinetic factors during film formation remains poorly understood.
